# Ectopic calcitonin-secreting pulmonary neuroendocrine tumor mimicking medullary thyroid carcinoma

**DOI:** 10.1210/jcemcr/luag194

**Published:** 2026-07-13

**Authors:** Riza Gökhan Baykal, Sefika Burcak Polat, Ekin Yigit Köroglu, Oya Topaloglu, Reyhan Ersoy, Bekir Cakir

**Affiliations:** Department of Endocrinology and Metabolism, Ankara Bilkent City Hospital, Ankara 06800, Türkiye; Department of Endocrinology and Metabolism, Ankara Yıldırım Beyazıt University Faculty of Medicine, Ankara Bilkent City Hospital, Ankara 06800, Türkiye; Department of Endocrinology and Metabolism, Ankara Bilkent City Hospital, Ankara 06800, Türkiye; Department of Endocrinology and Metabolism, Ankara Yıldırım Beyazıt University Faculty of Medicine, Ankara Bilkent City Hospital, Ankara 06800, Türkiye; Department of Endocrinology and Metabolism, Ankara Yıldırım Beyazıt University Faculty of Medicine, Ankara Bilkent City Hospital, Ankara 06800, Türkiye; Department of Endocrinology and Metabolism, Ankara Yıldırım Beyazıt University Faculty of Medicine, Ankara Bilkent City Hospital, Ankara 06800, Türkiye

**Keywords:** calcitonin, neuroendocrine tumor, ectopic hormone secretion, pulmonary neuroendocrine neoplasm, medullary thyroid carcinoma

## Abstract

Calcitonin-secreting neuroendocrine neoplasms of the lung are extremely rare. Differentiating medullary thyroid carcinoma (MTC) from ectopic calcitonin secretion is challenging. Elevated calcitonin usually triggers a thyroid-focused workup, but ectopic secretion must be considered to avoid misdiagnosis and unnecessary thyroidectomy. We report a 54-year-old man presenting with weight loss and back pain. Serum calcitonin was markedly elevated at 1011 pg/mL (SI: 293 pmol/L) (reference range, <16 pg/mL [SI: <4.67 pmol/L] for basal levels in men). Thyroid ultrasonography showed a >2 cm nodule in the right thyroid lobe. Fine-needle aspiration cytology revealed atypia of undetermined significance, and calcitonin washout was negative. 18F-fluorodeoxyglucose positron emission tomography showed intense uptake in a 4.5 cm right lower lobe pulmonary mass and a 6.5 cm right adrenal mass, with no thyroid uptake. Histopathological analysis of the lung lesion confirmed high-grade pulmonary neuroendocrine carcinoma. Systemic chemotherapy was initiated, but the patient demonstrated disease progression and died from respiratory failure. This case illustrates how ectopic calcitonin production can mimic MTC. Comprehensive evaluation with functional imaging and immunohistochemistry is essential for accurate diagnosis. Multidisciplinary collaboration is crucial in distinguishing thyroid malignancy from ectopic hypercalcitoninemia.

## Introduction

Calcitonin is a peptide hormone consisting of 32 amino acids, mainly secreted by the parafollicular C cells of the thyroid gland. It has long been considered a reliable and specific biochemical marker for medullary thyroid carcinoma (MTC) [1]. In clinical settings, a significant rise in serum calcitonin usually raises suspicion of MTC, leading to further assessment through thyroid imaging and fine-needle aspiration cytology (FNAC). Nevertheless, recent evidence highlights that elevated calcitonin levels are not exclusively indicative of MTC. Several physiological and pathological conditions, including chronic kidney disease, autoimmune thyroiditis, and smoking, can cause mild to moderate increases in calcitonin [2, 3]. In rare instances, neuroendocrine tumors outside the thyroid may also produce calcitonin ectopically, creating substantial diagnostic challenges [4].

A comprehensive evaluation of hypercalcitoninemia includes imaging modalities such as ultrasound, computed tomography (CT), and 18F-fluorodeoxyglucose positron emission tomography (18F-FDG PET), along with laboratory tests including calcitonin washout, carcinoembryonic antigen (CEA), and plasma metanephrines. Critical distinguishing factors involve the detection of calcitonin washout from thyroid nodules, the presence or absence of somatostatin receptor uptake, and histopathologic indicators such as the Ki-67 proliferation index, neuroendocrine marker expression, and thyroglobulin staining profile [5].

Pulmonary neuroendocrine neoplasms (NENs) represent an uncommon yet clinically significant consideration in the differential diagnosis. Although small-cell and large-cell neuroendocrine carcinomas of the lung more commonly produce alternative bioactive peptides, isolated cases of calcitonin-secreting pulmonary NENs have been documented. Some of these tumors can closely resemble MTC in both biochemical profile and immunohistochemical staining. Differentiating these entities is crucial, as it directly guides management. Misattribution of ectopic calcitonin to a thyroid origin may lead to unnecessary thyroidectomy [6].

We report ectopic hypercalcitoninemia in a 54-year-old man due to a high-grade pulmonary neuroendocrine carcinoma. The coexistence of markedly elevated calcitonin and thyroid nodules initially suggested MTC, highlighting an important diagnostic pitfall. This case underscores the need for a careful multidisciplinary approach to distinguish ectopic from thyroid-derived calcitonin and avoid unnecessary surgery.

## Case presentation

A 54-year-old man with no significant medical history presented with a 2-month history of progressive weight loss, nausea, intermittent vomiting, and back pain. He reported no smoking, medication use, or comorbidities, and had no family history of thyroid or neuroendocrine malignancies. Empirical antibiotics had been initiated at a primary care clinic for presumed gastroenteritis.

## Diagnostic assessment

Laboratory tests revealed elevated C-reactive protein (CRP) and procalcitonin levels. Given the associated weight loss, abdominal imaging was performed. Ultrasonography identified a nodular lesion measuring ∼52 mm in the right adrenal region, extending from the upper pole of the right kidney into the adrenal gland. The patient was referred to a tertiary care hospital, where he was evaluated by general surgery and subsequently referred to our service for further assessment. A noncontrast abdominal CT scan was performed to further characterize the adrenal lesion, and a comprehensive hormonal workup was initiated. Biochemical evaluation, including plasma and urinary fractionated metanephrines and normetanephrines, serum cortisol, adrenocorticotropic hormone (ACTH), and a 1 mg overnight dexamethasone suppression test, was within normal limits (see Table 1). Given the history of hypertension, the aldosterone-to-renin ratio was also measured. A chest radiograph showed an opacity in the right hemithorax, raising concern for a space-occupying lesion. Accordingly, thoracic imaging was performed.

**Table 1:** Patient laboratory investigations upon admission

Investigation	Result (reference range)	
	Conventional units	Système international (SI) units
Glucose	77 mg/dL (70-99 mg/dL)	4.3 mmol/L (3.9-5.5 mmol/L)
Creatinine	0.83 mg/dL (0.7-1.3 mg/dL)	73.4 µmol/L (61.9-114.9 µmol/L, for men)
Sodium	140 mEq/L (136-142 mEq/L)	140 mmol/L (136-142 mmol/L)
Potassium	4.2 mEq/L (3.5-5 mEq/L)	4.2 mmol/L (3.5-5 mmol/L)
TSH	4 mIU/L (0.55-4.78 mIU/L)	4 mIU/L (0.55-4.78 mIU/L)
sT4	1.07 ng/dL (0.89-1.76 ng/dL)	13.8 pmol/L (11.45-22.65 pmol/L)
sT3	2.97 pg/mL (2.3-4.2 pg/mL)	4.56 pmol/L (3.53-6.45 pmol/L)
Anti-TPO	39 IU/mL (<60 IU/mL)	
Anti-TG	<1.3 IU/mL (≤4.0 IU/mL)	1.3 kIU/L (≤4.0 kIU/L)
Thyroglobulin	229 ng/mL (1.6-60 ng/mL)	229 µg/L (1.6-60 µg/L)
Calcitonin	1011 pg/mL (<16 pg/mL)	293 pmol/L (<4.67 pmol/L, for men)
Procalcitonin	45.7 µg/L (<0.16 µg/L)	45.7 µg/L (<0.16 µg/L)
CEA	3.8 ng/mL (<2.5 ng/mL)	3.8 µg/L (<2.5 µg/L)
ACTH	17.1 pg/mL (10-60 pg/mL)	3.77 pmol/L (2.2-13.2 pmol/L)
Cortisol	23.2 µg/dL (5-25 µg/dL)	640 nmol/L (137.9-689.7 nmol/L)
DHEA-S	132.73 µg/dL (35-179 µg/dL)	3.59 µmol/L (0.95-4.85 µmol/L, for men aged 50-59 years)
Plasma metanephrine	58.4 pg/mL (<90 pg/mL)	0.29 nmol/L (<0.46 nmol/L)
Plasma normetanephrine	119.6 pg/mL (<200 pg/mL)	0.65 nmol/L (<1.1 nmol/L)

Abbreviations: TSH, thyroid-stimulating hormone; sT4, free thyroxine; sT3, free triiodothyronine; Anti-TPO, anti-thyroid peroxidase; Anti-TG, anti-thyroglobulin; ACTH, adrenocorticotropic hormone; DHEA-S, dehydroepiandrosterone sulfate; CEA, carcinoembryonic antigen.

Repeat laboratory testing revealed persistently elevated CRP and markedly increased procalcitonin levels. Based on these findings, serum calcitonin was measured, revealing striking hypercalcitoninemia at 1011 pg/mL (SI: 293 pmol/L) (reference range, <16 pg/mL [SI: <4.67 pmol/L] for basal levels in men). CEA was 3.8 ng/mL (SI: 3.8 µg/L), and thyroid function tests were unremarkable. Given the profound calcitonin elevation, MTC was considered the working diagnosis. Neck ultrasonography revealed multiple abnormal lymph nodes in both the central compartment and bilateral lateral chains. The largest, located in the right level IV region, measured 1.6 cm and showed a rounded configuration, loss of the echogenic hilum, and increased intranodal vascularity (Fig. 1). FNAC of the thyroid nodule showed atypia of undetermined significance (AUS), while lymph node aspirates were nondiagnostic. Repeat biopsy was suspicious for malignancy (Bethesda V) with nuclear features of papillary thyroid carcinoma. Importantly, calcitonin washout from both the thyroid nodule and lymph nodes was negative, arguing against an intrathyroidal source.

**Figure 1:**
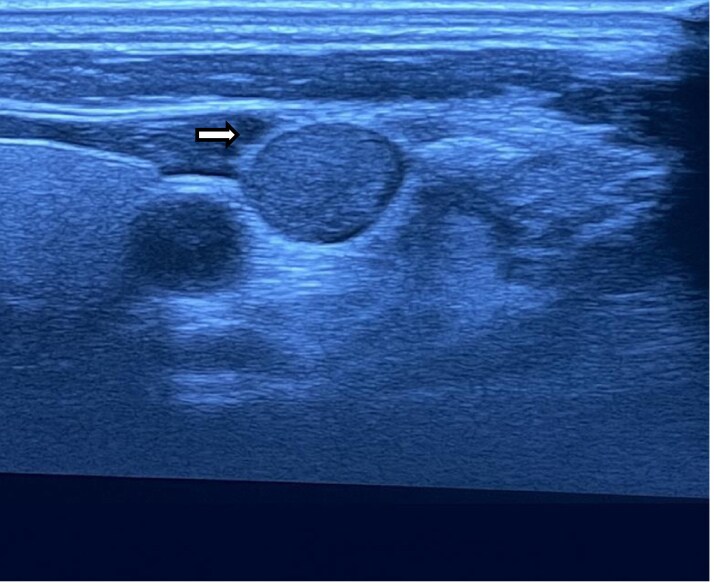
Ultrasound image demonstrating abnormal cervical lymphadenopathy in the left level IV region.

Abdominal CT revealed a 66 × 60 mm heterogeneously hypodense lesion with an attenuation of 30 Hounsfield units (HU) in the right adrenal region, suggestive of metastasis. The fat planes between the lesion and hepatic segment 6 were thinned, raising suspicion of invasion. In the left adrenal gland, multiple hyperdense nodules were observed, the largest measuring 16 × 14 mm. The presence of bilateral adrenal masses with attenuation values exceeding 20 HU on noncontrast CT strongly suggested extra-adrenal malignancy with possible adrenal metastases (Fig. 2). In the context of profound hypercalcitoninemia, several diagnostic considerations emerged. These included MTC with adrenal metastasis, although negative calcitonin washout argued against this; an independent primary malignancy as the ectopic source with adrenal involvement; or, less likely, coexisting PTC with a calcitonin-secreting pheochromocytoma. The possibility of ectopic secretion from a nonthyroidal malignancy or assay interference was also considered. Overall, these findings highlighted the diagnostic complexity of the case.

**Figure 2:**
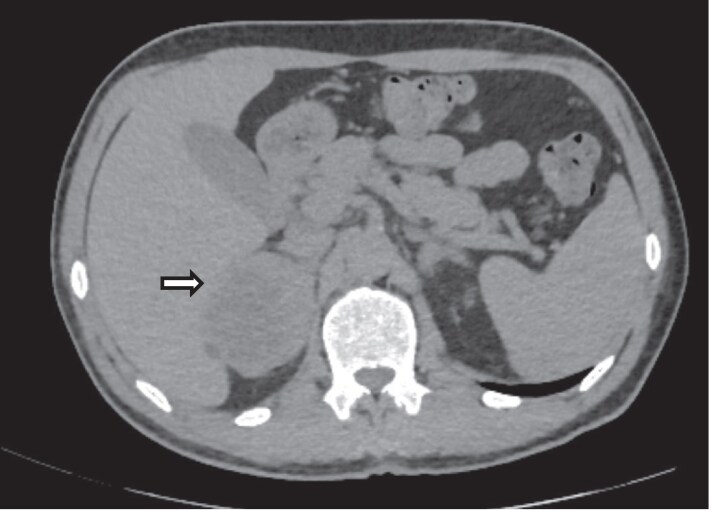
Abdominal CT shows a heterogeneously hypodense nodular lesion in the right adrenal gland, measuring ∼66 × 60 mm in axial plane with a mean density of 30 HU, suggestive of metastasis. The fat planes between the lesion and liver segment 6 appear markedly attenuated (suspicious for invasion). The left adrenal gland contains multiple hyperdense nodular lesions, the largest measuring 16 × 14 mm (also suggestive of metastasis).

Thoracic CT revealed a 43 × 42 mm spiculated mass in the peribronchovascular region of the right lower lobe. Subsequent 18F-FDG PET/CT demonstrated increased uptake in a 20 mm midline cerebellar lesion (maximum standardized uptake value [SUVmax]: 22.27). Brain magnetic resonance imaging was subsequently performed and confirmed the presence of a cerebellar lesion consistent with metastatic disease. No pathological FDG uptake was observed in the 15 mm hypodense nodule of the right thyroid lobe. In contrast, marked FDG uptake was detected in a 45 × 40 mm right lower lobe mass (SUVmax: 14.39), a 64 × 57 mm right adrenal mass (SUVmax: 10.77), and a 13 mm left adrenal nodular lesion (SUVmax: 7.84) (Fig. 3). To further evaluate the possibility of a neuroendocrine tumor, a 68Ga-DOTATATE PET scan was obtained simultaneously; however, no pathological uptake was demonstrated in either the pulmonary or adrenal lesions.

**Figure 3:**
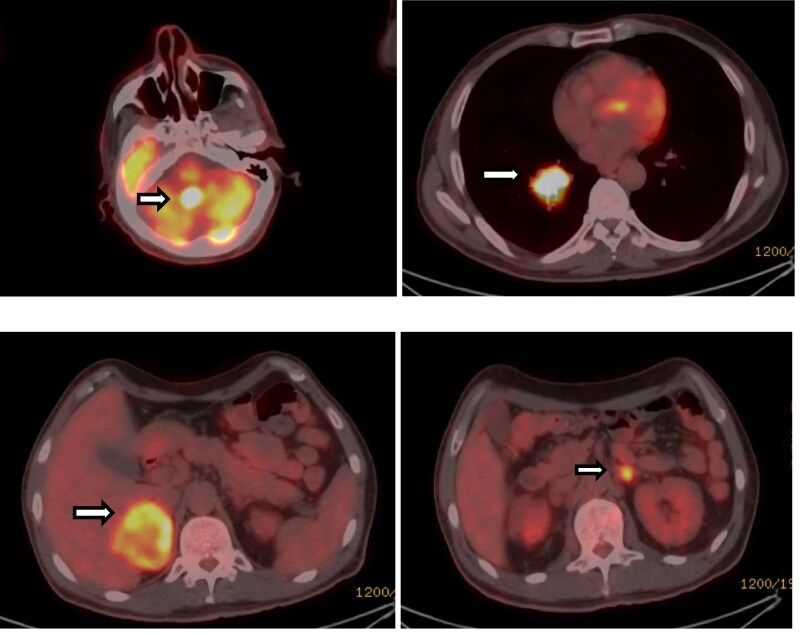
18F-fluorodeoxyglucose positron emission tomography demonstrating hypermetabolic lesions in the right lung, cerebellum, and both adrenal glands, consistent with a primary pulmonary malignancy and metastatic disease.

CT-guided biopsy of the pulmonary mass revealed a poorly differentiated carcinoma with neuroendocrine morphology. Immunohistochemistry showed strong positivity for CD56, synaptophysin, and calcitonin, while chromogranin A and napsin A were negative. The Ki-67 index was 70%, confirming high-grade pulmonary neuroendocrine carcinoma. The adrenal lesion was considered metastatic based on its radiologic features and 18F-FDG PET avidity, without tissue confirmation.

## Treatment

Following diagnosis, the patient was referred to medical oncology and started on platinum-based chemotherapy (cisplatin and etoposide) in September 2024. Given the inoperable disease and limited life expectancy, further evaluation of the thyroid nodules and cervical lymphadenopathy was deferred.

## Outcome and follow-up

After 3 cycles of cisplatin and etoposide chemotherapy, the patient developed profound muscle weakness and progressive functional decline, ultimately becoming unable to ambulate without assistance. A follow-up 18F-FDG PET scan performed 3 months later demonstrated disease progression, with persistence of metabolic activity despite therapy. Given the refractory nature of the malignancy, systemic chemotherapy was discontinued and the patient was transitioned to palliative care. He succumbed to respiratory failure in March 2025.

## Discussion

While hypercalcitoninemia is classically linked to MTC, markedly elevated levels may also arise from ectopic secretion by extrathyroidal NENs, which can closely resemble MTC both biochemically and histologically [4, 5].

Pulmonary NENs are recognized sources of ectopic calcitonin secretion. Neuroendocrine cells in the lung can produce bioactive peptides, and up to one-quarter of lung carcinomas may exhibit neuroendocrine differentiation and paraneoplastic syndromes [5, 7, 8].

Calcitonin secretion has been reported in various pulmonary settings, including lung cancer and smoking-related conditions. In small-cell lung cancer, elevated calcitonin may occur despite negative immunohistochemical staining, suggesting incomplete neuroendocrine differentiation [9]. A systematic review by Llewellyn et al reported lung NENs, including carcinoids and small- and large-cell carcinomas, presenting with elevated calcitonin levels [5]. In several cases, histopathologic resemblance to MTC resulted in misdiagnosis and unwarranted thyroidectomy. Similarly, in our patient, the coexistence of a thyroid nodule and markedly elevated calcitonin initially raised strong suspicion for MTC. However, negative calcitonin washout and absent thyroid FDG uptake were key in identifying an extrathyroidal source. Although calcitonin has been associated with secretory diarrhea, this symptom is not universally present. Its occurrence likely depends on tumor burden, duration of hormone exposure, and cosecretion of other bioactive peptides.

In cases resembling ours, several distinguishing features may assist in differentiating ectopic calcitonin-secreting neoplasms from MTC. Calcitonin washout from thyroid nodules is generally positive in MTC but tends to be negative when the source is extrathyroidal [5]. Functional imaging, such as 18F-FDG PET, often reveals intense metabolic activity at the ectopic site, while the thyroid remains inactive. Immunohistochemical findings may be less definitive, as both MTC and ectopic NENs frequently express calcitonin and neuroendocrine markers, including synaptophysin and chromogranin A; negative chromogranin A staining can occur in poorly differentiated neuroendocrine carcinomas, reflecting incomplete neuroendocrine differentiation. The presence or absence of thyroglobulin and TTF-1 expression may aid in determining the site of origin. Additionally, CEA levels, which are often elevated in MTC, may remain within normal limits in ectopic calcitonin-secreting tumors, as observed in our patient; however, CEA is not a diagnostic marker and should be interpreted only as a supportive finding.

In our case, the adrenal lesion, although not subjected to biopsy, was most consistent with metastatic involvement, given its intense metabolic activity and association with an aggressive pulmonary neuroendocrine carcinoma. The absence of 68Ga-DOTATATE uptake despite intense FDG activity likely reflects the high-grade nature of the tumor, as poorly differentiated neuroendocrine carcinomas often exhibit reduced somatostatin receptor expression but increased glucose metabolism. Of further significance, the Ki-67 proliferation index was 70%, supporting the diagnosis of a high-grade neuroendocrine carcinoma with an unfavorable prognosis. Despite initiation of platinum-based chemotherapy, the disease progressed rapidly, in keeping with reports that calcitonin-secreting pulmonary neuroendocrine carcinomas often follow an aggressive clinical course and exhibit limited responsiveness to available therapies. In high-grade pulmonary neuroendocrine carcinomas, systemic chemotherapy is the mainstay of treatment due to the aggressive nature of the disease and frequent presence of metastatic involvement at diagnosis, whereas surgical intervention is typically reserved for localized disease.

Our case provides several instructive lessons for clinicians. First, without the measurement of procalcitonin during the evaluation of a suspected infection, serum calcitonin might not have been evaluated, despite the presence of an adrenal mass. Second, a pulmonary lesion on chest radiograph with bilateral adrenal masses should raise suspicion for metastatic disease and prompt timely evaluation. Third, elevated calcitonin with a thyroid nodule and suspicious lymphadenopathy does not necessarily indicate MTC. Fine-needle aspiration with calcitonin washout should be performed before surgery to avoid misdiagnosis. This case adds to the existing literature by highlighting an unusual diagnostic pathway initiated by elevated procalcitonin levels, the coexistence of thyroid nodules mimicking MTC, and the critical role of calcitonin washout in identifying an extrathyroidal source. In addition, the discordant FDG and DOTATATE imaging findings provide further insight into the biology of high-grade neuroendocrine carcinomas.

## Learning points

- Markedly elevated plasma calcitonin is pathognomonic but not diagnostic of MTC and may originate from ectopic secretion by extrathyroidal NENs.
- Negative calcitonin washout from thyroid nodules and lymph nodes is a critical clue arguing against a thyroidal source of hypercalcitoninemia.
- Functional imaging with 18F-fluorodeoxyglucose PET can help localize ectopic calcitonin-secreting tumors when thyroid imaging is inconclusive.
- Pulmonary neuroendocrine carcinomas, although rare, should be considered in the differential diagnosis of unexplained hypercalcitoninemia, particularly in the presence of adrenal masses.
- A multidisciplinary and stepwise diagnostic approach is essential to avoid misdiagnosis and unnecessary thyroid surgery in patients with ectopic calcitonin secretion.

## Contributors

All authors made individual contributions to authorship. R.G.B. and S.B.P. were involved in the clinical evaluation, diagnosis, management, and follow-up of the patient. E.Y.K. contributed to the conception of the case report and interpretation of clinical findings. O.T., R.E., and B.C. contributed to the clinical management of the patient, diagnostic evaluation, and interpretation of findings. All authors reviewed and approved the final manuscript and agree to be accountable for all aspects of the work.

## Data Availability

Original data generated and analyzed during this study are included in this published article.
